# AAPH or Peroxynitrite-Induced Biorelevant Oxidation of Methyl Caffeate Yields a Potent Antitumor Metabolite

**DOI:** 10.3390/biom10111537

**Published:** 2020-11-11

**Authors:** Laura Fási, Ahmed Dhahir Latif, István Zupkó, Sándor Lévai, Miklós Dékány, Zoltán Béni, Árpád Könczöl, György Tibor Balogh, Attila Hunyadi

**Affiliations:** 1 Institute of Pharmacognosy, Interdisciplinary Excellence Centre, University of Szeged, Eötvös str. 6, H-6720 Szeged, Hungary; fasi.laura@pharmacognosy.hu (L.F.); latif.ahmed@pharmacognosy.hu (A.D.L.); 2 Department of Pharmacodynamics and Biopharmacy, University of Szeged, Eötvös str. 6, H-6720 Szeged, Hungary; zupko@pharm.u-szeged.hu; 3 Department of Chemistry, Gedeon Richter Plc., Gyömrői u. 19-21, H-1103 Budapest, Hungary; s.levai@richter.hu (S.L.); m.dekany@richter.hu (M.D.); z.beni@richter.hu (Z.B.); arpad.konczol@rotachrom.com (A.K.); 4 Interdisciplinary Centre for Natural Products, University of Szeged, Eötvös str. 6, H-6720 Szeged, Hungary

**Keywords:** antioxidant, hydroxycinnamate, methyl *p*-coumarate, methyl caffeate, lignan, reactive oxygen and nitrogen species, peroxynitrite, oxidative stress, scavengome, diversity-oriented synthesis, drug discovery

## Abstract

Hydroxycinnamic acids represent a versatile group of dietary plant antioxidants. Oxidation of methyl-*p*-coumarate (**pcm**) and methyl caffeate (**cm**) was previously found to yield potent antitumor metabolites. Here, we report the formation of potentially bioactive products of **pcm** and **cm** oxidized with peroxynitrite (ONOO¯), a biologically relevant reactive nitrogen species (RNS), or with α,α′-azodiisobutyramidine dihydrochloride (AAPH) as a chemical model for reactive oxygen species (ROS). A continuous flow system was developed to achieve reproducible in situ ONOO¯ formation. Reaction mixtures were tested for their cytotoxic effect on HeLa, SiHa, MCF-7 and MDA-MB-231 cells. The reaction of **pcm** with ONOO¯ produced two fragments, an o-nitrophenol derivative, and a new chlorinated compound. Bioactivity-guided isolation from the reaction mixture of **cm** with AAPH produced two dimerization products, including a dihydrobenzofuran lignan that exerted strong antitumor activity in vitro, and has potent in vivo antimetastatic activity which was previously reported. This compound was also detected from the reaction between **cm** and ONOO¯. Our results demonstrate the ROS/RNS dependent formation of chemically stable metabolites, including a potent antitumor agent (**5**), from hydroxycinnamic acids. This suggests that diversity-oriented synthesis using ROS/RNS to obtain oxidized antioxidant metabolite mixtures may serve as a valid natural product-based drug discovery strategy.

## 1. Introduction

Hydroxycinnamic acids are widespread and abundant dietary antioxidants that are present in many foodstuffs [1]. A broad scale of beneficial bioactivities, including chemo-preventive and antitumor effects, has been reported for members of this compound group, and their well-known antioxidant properties have been implied to play a role in such bioactivities [2,3,4].

In our recent review, we suggested that it may be a relevant, yet unexplored drug discovery strategy to perform systematic studies on chemically oxidized mixtures obtained from the biomimetic oxidation of small molecule antioxidants [5]. This is based on the notion that i) scavenging reactive oxygen or nitrogen species (ROS or RNS, respectively, collectively referred to as RONS) with antioxidants frequently results in chemically stable, bioactive, oxidized metabolites, ii) such metabolites may have a dramatically altered bioactivity compared to their parent compound, and iii) this altered bioactivity is due to new chemical information directly translated from oxidative stress. Therefore, this chemical metabolite space, that we defined as the scavengome, should be particularly rich in bioactive compounds [5].

Diversity-oriented synthesis is a major concept in drug discovery [6,7,8], and it is currently in a paradigm shift towards biological performance diversity instead of plain chemical diversity [9]. Based on the above, it may be a reasonable strategy to target biological diversity by utilizing RONS-mediated oxidation as a driving principle for an antioxidant-inspired biorelevant expansion of chemical space. Such an approach may be particularly interesting for antitumor drug discovery, which is full of controversy concerning the real therapeutic value of “double-edged sword” antioxidants that have long been a subject of intense debate [10,11,12,13].

Due to their simple chemical structure and abundance as dietary antioxidants, the hydroxycinnamic acid derivatives methyl-*p*-coumarate (**pcm**) and methyl-caffeate (**cm**) may serve as good model compounds to related studies. As a first proof-of-concept, we obtained indirect evidence for the ROS scavenging-related in situ formation of a *p*-quinol derivative, graviquinone, from **pcm**. Graviquinone was identified as a promising antitumor lead compound due to its potent cytotoxic activity, good tumor selectivity, and ability to modulate DNA damage response through interfering with the activation of checkpoint kinases 1 and 2 [14]. Previously, it was reported that Ag_2_O catalyzed oxidation of **cm** yielded racemate of a dihydrobenzofuran lignan, which had antiproliferative activity several orders of magnitude stronger than that of **cm** on a variety of breast cancer cell lines [15]. Furthermore, the *2R*,*3R* enantiomer of this lignan acted as a potent antitubulin agent [15], and the same enantiomer was also reported to exert antiangiogenic activity [16]. Notably, racemate of this dihydrobenzofuran lignan was recently reported as a highly promising antimetastatic agent that exerts this activity in vivo through its interaction with the tumor microenvironment by inducing the interleukin-25 (IL-25) secretion of tumor-associated fibroblasts [17].

In the present work, our aim was to evaluate RONS scavenging-mediated formation of metabolite patterns from **pcm** and **cm**, and to search for antitumor compounds within the product mixtures. A fully biorelevant reagent, peroxynitrite (ONOO¯), was selected to study the effects of RNS on these two dietary antioxidants, and the peroxyl radical initiator α,α′-azodiisobutyramidine dihydrochloride (AAPH) was selected to study the ROS scavenging-related formation of oxidized metabolites.

## 2. Materials and Methods 

### 2.1. Continuous-Flow Preparation of Peroxynitrite and Its Reaction with pcm or cm 

**Figure 1:**
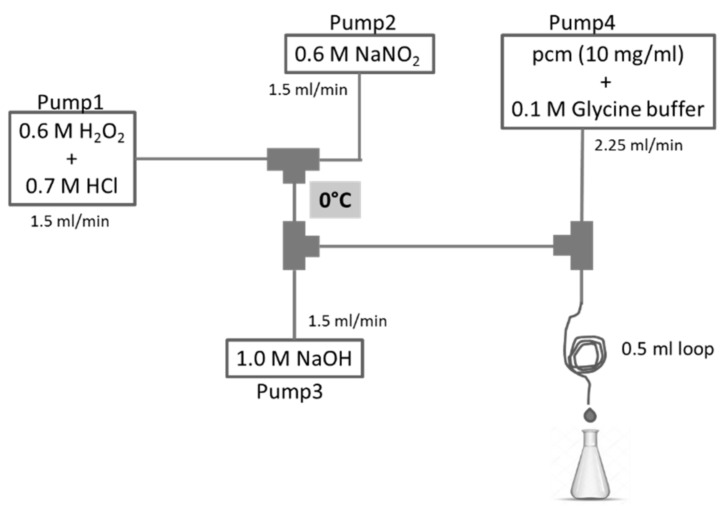
Experimental setup of the preparation of peroxynitrite and its reaction with the hydroxycinnamates of methyl-*p*-coumarate (**pcm)** or methyl-caffeate (**cm)**.

The experimental setup of the continuous-flow system in which the reactions with peroxynitrite were performed is shown in Figure 1. From Pump1, a mixture of 0.6 M H_2_O_2_ and 0.7 M HCl solution was pumped with a flow rate of 1.5 mL/min. From Pump2, a solution of 0.6 M NaNO_2_ was pumped with the same flow rate. These two channels were mixed in the first junction, then in the second junction 1.0 M NaOH solution was mixed in, that was pumped from Pump3 with a flow rate of 1.5 mL/min. From Pump4, a mixture of **pcm** or **cm** and 0.1 M glycine buffer was pumped, and it was mixed with peroxynitrite in the third junction. Here, the reaction took place instantaneously. At the end of a 0.5 mL loop the reaction mixture was collected in a chilled flask. All tubes were kept in an ice bath (0 °C).

### 2.2. Isolation of Products from the Reaction between pcm and Peroxynitrite

The reaction mixture was analyzed on an Agilent 1200 liquid chromatography system coupled with an Agilent 6410 triple quadrupole mass spectrometer equipped with an ESI source (Agilent Technology, Waldbronn, Germany). Analyses were carried out at 40 °C on a Cortecs (C18, 150 × 4.6, 2.7 µm) column with a mobile phase flow rate of 1.2 mL/min. Gradient elution was used by gradually increasing Solvent B (0.1% TFA in acetonitrile:water/95:5, *v/v*) in Solvent A (0.1% TFA in H_2_O) from 0% to 40% in 10 min, then to 55% from 10 to 11 min, and finally to 100% until 20 min had passed, then washed with 100% from 20 to 22 min, and decreased back to 0%.

The reaction mixture was purified by a Shimadzu LC-8 preparative HPLC on a gradient system of two LC-8A preparative pumps connected to an SPD M20A diode array detector. An SIL-10AP autosampler, an FRC-10A fraction collector, a CMB-20A communications bus module, and a CTO-20AC column oven were the parts of the system. A Chromolith C18 (100 × 4.6 mm, monolithic) column was used with a flow rate of 30 mL/min, at 40 °C. The appropriate solvent system was a gradient elution of acetonitrile in water, from 0 to 90% in 10 min, washed with 90% from 10 to 15 min, and back to 0 %. Compounds **3** (t_R_: 8.033 min) and **4** (t_R_: 11.233 min) were purified. The reaction was repeated to obtain higher amounts of the compounds, but the purification process needed to be modified because of small differences in the product profile. Two columns, a Chromolith and a Reprosil Chiral-Mix (100 × 4.6 mm, 5 µm) were connected one after the other. The solvent system, flow rate, and temperature were the same as above. Compounds **1** (t_R_: 10.519 min) and **2** (t_R_: 12.657) were purified. Before the purifications, the reaction mixture was dried under a nitrogen stream, dissolved in 10 mL of acetonitrile, filtered on syringe filter, re-dissolved in 1.5 mL of acetonitrile, and 200 or 300 µL of the mixture was injected. The collected fractions were lyophilized overnight. Isolated yields of compounds **1**–**4** from two separate reactions were 0.43 mg (0.35%), 0.59 mg (0.70%), 0.48 mg (3.6%), and 0.40 mg (2.9%), respectively.

### 2.3. Preparation of Crude Product Mixtures for Bioactivity Screening

Amounts of 50 mg of methyl *p*-coumarate (**pcm**) or methyl caffeate (**cm**) were reacted with peroxynitrite in a flow system as shown in Figure 1. Aliquots of 0.7 mL of each reaction mixture were dried under a nitrogen stream, dissolved in ethyl acetate (2 mL), and extracted with water (3 × 2 mL). The collected water phase was washed once with ethyl acetate (5 mL), and the organic phase was dried with anhydrous MgSO_4_, then dried with the nitrogen stream.

Solutions of **pcm** or **cm** were prepared at 10 mg/mL concentration (5 mg in 0.5 mL acetonitrile:water/1:1 or 9:1, or in MeOH:water/1:1), and reacted with a 10 mg/mL solution of AAPH dissolved in the same solvent. Due to solubility issues with AAPH, an additional 0.5 mL of water had to be added to the solutions in acetonitrile:water/9:1 before the reaction was started, therefore the concentrations and the solvent composition were modified accordingly. The reactions were heated at 60 °C in an ultrasonic bath for 4 h. Subsequently, all reaction mixtures were pre-purified for cytotoxic screening experiments as described above.

These samples were analyzed by LC-DAD-MS on a Cortecs (C18, 150 × 4.6 mm, 2.7 µm) column with a gradient elution of Solvent B (0.1% TFA in acetonitrile:water/95:5) in Solvent A (0.1% TFA in water) from 0 to 100% in 10 min, and washed with 100% from 10 to 12 min. Chromatographic fingerprints and cytotoxicity data for the active product mixtures obtained from **cm** are provided as Supplementary Materials, in Figures S1–S4 and Table S1.

### 2.4. Longitudinal Study of the Reaction between cm and AAPH 

An amount of 50 mg of **cm** was dissolved in a 5 mL mixture of acetonitrile:water (9:1, *v/v*). AAPH (100 mg) was dissolved in the same solvent system at 10 mg/mL concentration, and 2.5 mL of water was added to obtain a clear solution. The reaction was stirred and heated at 60 °C in an oil bath, and 1.4 mL of sample was taken at specified times. The first sample was taken out immediately after reagent was added (i.e., t_0_), and subsequent samples were taken at 1, 4, 8, 24, 30 and 48 h. The reaction was monitored by TLC, and dichloromethane:methanol (9.5:0.5, *v/v*) was used as a solvent system. As a visual observation, the color of the solution was yellow at the beginning and changed through orange to red.

Each sample was dried under a nitrogen stream, dissolved in ethyl acetate, then extracted with water (3 x 2 mL). The water phase was washed once with 5 mL of ethyl acetate. The organic phase was dried under the nitrogen stream. Each sample was subsequently tested for its cytotoxic activity on an HeLa cell line. Chromatographic fingerprints of the samples were taken by HPLC on a gradient system of two PU-2080 pumps connected to an MD-2010 Plus photodiode-array detector (Jasco Analytical Instruments, Tokyo, Japan), on a Kinetex Biphenyl (5 µm, 100 Å, 250 × 4.6 mm) column with the solvent system 30% acetonitrile in water to 70% acetonitrile. Each sample was also analyzed by supercritical fluid chromatography (SFC) on a Jasco semi-preparative chromatographic system (PU-4386 and PU-4086 pumps, AS-4350 SFC autosampler, CO4060 column oven, BP-4340 back-pressure regulator; Jasco Analytical Instruments, Tokyo, Japan), Luna Silica (2) column (5µm, 100 Å, 250 × 4.6 mm, Phenomenex, Torrance, CA, USA) with the solvent system CO_2_–EtOH (9:1, *v/v*), at t = 30 °C, p = 25 MPa, and flow rate of 2 mL/min.

### 2.5. Isolation of Products from the Reaction between cm and AAPH

A higher amount, 100 mg, of **cm** was dissolved in 10 mL of the mixture of acetonitrile:water (9:1). A quantity of 200 mg of AAPH was dissolved in 20 mL of the same solvent, then a further 5 mL water was added to overcome solubility problems. The reaction was stirred at 60 °C in an oil bath, and after 24 h the reaction was cooled down to room temperature, dried under a nitrogen stream, dissolved in ethyl acetate, and extracted with water (3 × 20 mL). The water phase was washed once with 30 mL of ethyl acetate. The organic phase was dried with anhydrous sodium sulfate, then evaporated under the nitrogen stream. Isolation was carried out by preparative HPLC, using the pump, detector and fraction collector of an Armen Spot CPC (Armen Instrument, Saint Ave, France) on a Kinetex biphenyl preparative HPLC column (5 µm, 100 Å, AX, 250 × 21.2 mm), with an isocratic elution of 36% acetonitrile (aq.), with a flow rate of 15 mL/min. Five fractions were purified, and their purity was subsequently tested by the above-described Jasco HPLC system on a Kinetex Biphenyl column (5 μm, 100 Å, 250 × 4.6 mm), with a flow rate of 1 mL/min. Each fraction was tested for cytotoxic effects, to allow the isolation of cytotoxic compounds other than the predicted active constituent, compound **5**. Repeated HPLC purification was necessary for the fraction containing mainly compound **5** (16.0 mg), using high loading of the Kinetex Biphenyl (5 μm, 100 Å, 250 × 4.6 mm) column with isocratic elution of 34% acetonitrile (aq.) with a flow rate of 1 mL/min. With this method, compound **5** (9.3 mg, 18.5%) was obtained at a purity of >95%. HPLC fraction 5 (7.4 mg) was purified by supercritical fluid chromatography (SFC) to obtain compound **6** (2.2 mg, 4.38%).

### 2.6. Structure Elucidation

NMR spectra were recorded at 25 °C on a Bruker 500 MHz spectrometer equipped with a TCI cryoprobe operating at 499.9 MHz for 1H and 125.7 MHz for ^13^C (Billerica, MA, USA). Chemical shifts were referenced to TMS or residual solvent signals (3.31 (^1^H)/49.15 ppm (^13^C) in the case of MeOH-d4, 77.0 ppm (^13^C) in the case of CDCl_3_, and 1.95(^1^H)/1.39 ppm (^13^C) in the case of acetonitrile-d3. Standard one- and two-dimensional spectra were collected in all cases using the pulse sequences available in the Bruker Topspin 3.5 sequence library. NMR assignments for all isolated compounds were in good agreement with the data reported earlier. 

HRMS and MS/MS analyses were performed on a Thermo Velos Pro Orbitrap Elite (Thermo Fisher Scientific) system. The ionization method was electron spray ionization (ESI) operating either in positive or negative ion mode. For MS/MS spectra, the protonated molecular ion peaks were fragmented by collision induced dissociation (CID) at a normalized collision energy of 35–45% using helium as the collision gas. The samples were dissolved in methanol prior the analysis. Both data acquisition and analysis were accomplished with Xcalibur software version 4.0 (Thermo Fisher Scientific).

Experimental details of the compounds prepared in this study are detailed below.

*p*-Hydroxybenzaldehyde (**1**). HRMS: M-H = 121.02872 (delta = −6.5 ppm; C_7_H_5_O_2_). HR-ESI-MS-MS (CID = 35%; rel. int. %): 93(100); 92(17). ^1^H NMR (499.9 MHz, CH_3_CN-d_3_) δ = 9.82 (1H, s, H-7), 7.70–7.84 (2H, m, H-6, 2), 6.90–7.00 (2H, m, H-5,3); ^13^C NMR (125.7 MHz, CH_3_CN-d_3_) δ = 116.9 (C-5,3), 130.5 (C-1), 133.1 (C-6,2), 163.8 (C-4), 191.8 (C-7).

*p*-Nitrophenol (**2**). HRMS: M-H = 138.01900 (delta = −4.8 ppm; C_6_H_4_O_3_N). HR-ESI-MS-MS (CID = 35%; rel. int. %): 108(100). ^1^H NMR (499.9 MHz, MeOH-d_4_) δ = 8.07–8.16 (1H, m, H-6,2), 6.83–6.92 (2H, m, H-5,3); ^13^C NMR (125.7 MHz, MeOH-d_4_) δ = 165.6 (C-4), 141.4 (C-1), 127.3 (C-6,2), 116.8 (C-5,3).

Compound **3**. HRMS: M + Na = 253.02334 (delta = −1.9 ppm; C_10_H_11_O_4_ClNa). HR-ESI-MS-MS (CID = 35%; rel. int. %): 217(100). ^1^H NMR (499.9 MHz, MeOH-d_4_) δ = 7.17–7.27 (2H, m, H-6,2), 6.71–6.80 (2H, m, H-5,3), 4.82 (3H, d, *J* = 8.9 Hz, H-7), 4.36 (1H, d, *J* = 8.9 Hz, H-8), 3.80 (3H, s, H-10); ^13^C NMR (125.7 MHz, MeOH-d_4_) δ = 53.0 (C-10), 60.7 (C-8), 75.9 (C-7), 115.8 (C-5,3), 129.5 (C-6,2), 132.0 (C-1), 158.1 (C-4), 170.7 (C-9).

Compound **4.** 3-Nitro-*p*-coumaric acid methyl ester. HRMS: M-H = 222.03917 (delta = −2.4 ppm; C_10_H_8_O_5_N). HR-ESI-MS-MS (CID = 45%; rel. int. %): 192(100); 190(20); 177(17); 163(18); 133(11). ^1^H NMR (500 MHz, MeOH-d_4_) δ = 8.27 (1H, d, *J* = 2.1 Hz, H-2), 7.88 (1H, dd, *J* = 8.8 Hz, *J* = 2.1 Hz, H-6), 7.67 (1H, d, *J* = 16.0 Hz, H-7), 7.16 (1H, d, *J* = 8.8 Hz, H-5), 6.51 (1H, d, *J* = 16.0 Hz, H-8), 3.79 (3H, s, H-10); ^13^C NMR (125.7 MHz, MeOH-d_4_) δ = 169.1 (C-9), 157.5 (C-4), 143.9 (C-7), 136.5 (C-3), 136.2 (C-6), 127.7 (C-1), 126.9 (C-2), 122.2 (C-5), 52.4 (C-10).

Compound **5**. HRMS: M + H = 387.10742 (delta = −0.1 ppm; C_20_H_19_O_8_). HR-ESI-MS-MS (CID = 45%; rel. int. %): 355(100); 323(81). ^1^H NMR (500 MHz, CHCl_3_-d) δ = 7.60 (1H, d, *J* = 15.9 Hz, H-7), 7.12 (1H, br s, H-6), 7.06 (1H, d, *J* = 1.3 Hz, H-2), 6.90 (1H, d, *J* = 1.3 Hz, H-2’), 6.84–6.87 (2H, m, H-5’, 6’), 6.29 (1H, d, *J* = 15.9 Hz, H-8), 6.09 (1H, d, *J* = 7.6 Hz, H-7’), 4.31 (21H, d, *J* = 7.6 Hz, H-8’), 3.83 (3H, s, H-10’), 3.80 (3H, s, H-10); ^13^C NMR (125.7 MHz, CHCl_3_-d) δ = 170.6 (C-9’), 167.7 (C-9), 148.2 (C-4), 144.6 (C-7), 144.0 (C-4’), 143.8 (C-3’), 140.3 (C-3), 132.5 (C-1’), 129.1 (C-1), 125.3 (C-5), 119.0 (C-6’), 117.6 (C-6), 116.0 (C-2), 115.9 (C-8), 115.6 (C-5’), 113.1 (C-2’), 87.2 (C-7’), 55.8 (C-8’), 53.0 (C-10’), 51.7 (C-10).

Compound **6**. HRMS: M + H = 387.10703 (delta = −1.1 ppm; C_20_H_19_O_8_). HR-ESI-MS-MS (CID = 45%; rel. int. %): 355(100); 345(42); 323(5); 313(5); 277(5); 193(25). ^1^H NMR (500 MHz, MeOH-d_4_) δ = 7.59 (1H, d, *J* = 15.8 Hz, H-7), 7.21 (1H, d, *J* = 2.1 Hz, H-2), 7.17 (1H, dd, *J* = 8.3 Hz, *J* = 2.0 Hz, H-6), 6.98 (1H, d, *J* = 8.3 Hz, H-5), 6.81 (1H, d, *J* = 7.2 Hz, H-2’), 6.75 (1H, d, *J* = 7.9 Hz, H-5’), 6.71 (1H, dd, *J* = 7.9 Hz, *J* = 2.1 Hz, H-6’), 6.39 (1H, d, *J* = 15.8 Hz, H-8), 5.09 (1H, d, *J* = 5.8 Hz, H-7’), 4.86* (1H, m, H-8’), 3.76 (3H, s, H-10), 3.63 (3H, s, H-10’), (*from HSQC, overlaps with residual water signal); ^13^C NMR (126 MHz, MeOH-d_4_) δ = 169.7 (C-9’), 169.4 (C-9), 147.4 (C-4’), 146.1 (C-4), 145.9 (C-7), 144.8 (C-3), 144.1 (C-3’), 130.1 (C-1), 128.2 (C-1’), 123.7 (C-6), 120.1 (C-6’), 118.6 (C-5), 118.1 (C-2), 117.3 (C-8), 116.4 (C-5’), 115.4 (C-2’), 78.1 (C-8’), 77.4 (C-7’), 53.1 (C-10’), 52.2 (C-10).

### 2.7. Cell Lines

The human gynecological cancer cell lines isolated from breast cancers (MCF7 and MDA-MB-231), and cervical adenocarcinoma (HeLa) were obtained from The European Collection of Cell Cultures, Salisbury, U.K., while the cervical carcinoma (SiHa) was purchased from the American Type Tissue Culture Collection, Manassas, Virginia, USA. All the cells were maintained in Minimum Essential Medium that was supplemented with 10% fetal bovine serum, 1% non-essential amino acids, and 1% penicillin-streptomycin at 37 °C in a humidified atmosphere condition. All media and supplements were purchased from Lonza Group Ltd. (Basel, Switzerland).

### 2.8. Cell Viability Testing

The cytotoxic properties of the prepared compounds were determined in human adherent gynecological cancer cell lines by the MTT [3-(4,5-dimethylthiazol-2-yl)-2,5-diphenyltetrazolium bromide] assay as published before [18]. Briefly, all cell lines were plated at a density of 5000 cells/well in 96 well plates. After 24 h, 100 µL of new media containing the test samples at 0.02, 0.05, 0.1, 0.2, 0.4, 0.9, 1.8, 3.7, 7.5, or 15 µM concentration was added and incubated for 72 h under cell-culturing conditions. The living cells were assayed by the addition of 44 µL of MTT solution (5 mg/mL) and incubated for a further 4h. After we removed the medium from the wells by aspiration, the precipitated formazan crystals were dissolved by adding 100 µL of dimethyl sulfoxide to each well and shaking the plate at 37 °C for 1h. The absorbance was measured at 545 nm with a microplate reader. Cisplatin, a clinically used anticancer agent, was used as a positive control. The sigmoidal dose–response curves were fitted to determine the fifty percent inhibitory concentrations (IC_50_ values) by using the nonlinear regression model log (inhibitor) vs. normalized response and variable slope fit of the GraphPad Prism 5.01 software (GraphPad Software Inc., San Diego, CA, USA). The in vitro experiments were performed in two independent experiments with at least five parallel wells. The IC_50_ value of the oxidative stress-inducing *tert*-butyl hydroperoxyde (*t*-BHP) was determined in the same way. Whenever product mixtures or fractions were tested, the concentrations were calculated with the molecular mass of the parent compound (**pcm** or **cm**) so that such IC_50_ values were expressed as parent compound equivalents.

### 2.9. Testing the Cytotoxicity of cm with or without the Presence of t-BHP-Induced Oxidative Stress

To assess the effect of oxidative stress on the cytotoxicity of **cm**, each cell line was tested in six groups, i.e., three cell controls and three for **cm** treatment. Each group was plated as described above, and treated for 24h with medium only, medium containing *t*-BHP at its 1/3 IC_50_ concentration, or medium containing *t*-BHP at its IC_50_^(72h)^ concentration. Then, the medium was removed, the cells were washed with PBS, and a new medium was added, with or without **cm** treatment. The cells were further incubated for 72 h and subsequently tested for viability by MTT assay as described above. For each treatment, the corresponding cell control underwent the same handling. Statistical analysis of the results was performed by two-way analysis of variance (ANOVA), followed by a Bonferroni post-hoc test, and differences were considered significant at *p* < 0.05.

## 3. Results and Discussion

### 3.1. In Situ Continuous-Flow Biomimetic Reaction Application of Peroxynitrite as an Oxidative Agent

Despite its short half-life (ca. 10–20ms), peroxynitrite can cross biological membranes and diffuse across up to two cell diameters [19], therefore it is a relevant biological oxidant in terms of being scavenged by small-molecule antioxidants. In the first part of our study, we aimed to set up well-controlled reaction conditions between **pcm** or **cm** and peroxynitrite (ONOO¯). To achieve this, a continuous flow reaction (CFR) system was constructed, in which ONOO¯ could be prepared in situ from nitrite and hydrogen peroxide, similarly to the method of Robinson and Beckman [20]. Schematic representation of the CFR system is presented in Figure 1.

**Figure 2:**
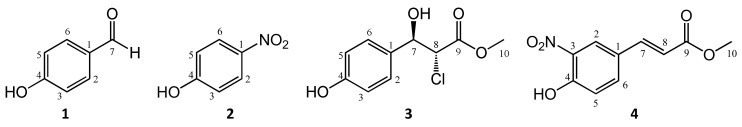
Structures of the metabolites **1**–**4** obtained from the reaction between **pcm** and peroxynitrite. For the racemic compound **3** only one enantiomer is shown for clarity.

To test the CFR method as a preparative tool, methyl *p*-coumarate (**pcm**) was first reacted with ONOO¯ in the above system. By using preparative HPLC, four products (**1**–**4**) were isolated from the reaction mixtures obtained this way. The structures of these compounds were elucidated by high-resolution mass spectroscopy (HRMS) and comprehensive 1- and 2D nuclear magnetic resonance (NMR) spectroscopic methods, and they are presented in Figure 2. Compound **3** is reported here for the first time.

The NMR chemical shifts were in good agreement with those published in the literature for compounds **1 [21]**, **2 [22]**, and **4** [23], while according to our literature search, compound **3** has not yet been reported. However, stereoisomers of the structurally highly similar 4-OMe derivative have been the subject of many investigations [24,25,26]. Our NMR data are in good agreement with those reported by Matsuki et al. [24]. Based on the findings reported by Odile et al. [27], and the observed *J* = 8.9 Hz coupling constant between H7 and H8, compound **3** is the racemic mixture of the *anti* isomers depicted in Figure 2.

The formation of *p*-hydroxybenzaldehyde (**1**) from **pcm** was also observed in our previous study on its reaction with PIFA or ˙OH radicals [14], and a similar fragmentation and subsequent radical reaction of the aromatic ring with ONOO¯ would intuitively explain the formation of *p*-nitrophenol (**2**). The somewhat unexpected formation of compound **3** must be the result of a secondary reaction between a reactive intermediate of **pcm**, oxidized at the trans-olefin moiety, and a chloride ion that is a contaminant of ONOO¯ prepared this way (see Figure 1). However, contaminant in this case or not, chloride is a major electrolyte present in the intra- and extracellular fluid. Therefore, its presence further strengthens the appropriateness of the present experimental setup to study the chemical space of metabolites that may be formed upon ONOO¯ scavenging in a biological environment. Finally, compound **4** represents a good example for the ability of ONOO¯ to act as a nitrating agent, not only an oxidant. Nitration of biological macromolecules by ONOO¯ is an important mechanism in redox signaling (mainly through nitrolipids) and oxidative damage (mainly through nitroproteins) [28]. The formation of compound **4** shows that an irreversible structural change through nitration of a small-molecule antioxidant, such as **pcm**, can also take place when that antioxidant scavenges ONOO¯.

### 3.2. Search for Antitumor Metabolites of pcm and cm Oxidized by Peroxynitrite or AAPH

In the second part of our study, we aimed to find ROS/RNS scavenging-related oxidized metabolites of **pcm** or **cm** through reacting them with ONOO¯ or AAPH. Firstly, crude reaction mixtures were prepared and tested for their cytotoxic activity on human gynecological cancer cell lines (HeLa, SiHa, MCF-7 and MDA-MB-231). In this study, none of the reaction mixtures of **pcm** showed an increased cytotoxic effect. On the other hand, a greatly increased cytotoxic effect was observed when **cm** was reacted with AAPH, and this effect was by far the strongest on HeLa cells (see Supplementary Materials, Table S1). Therefore, this reaction was selected for further studies.

**Figure 3:**
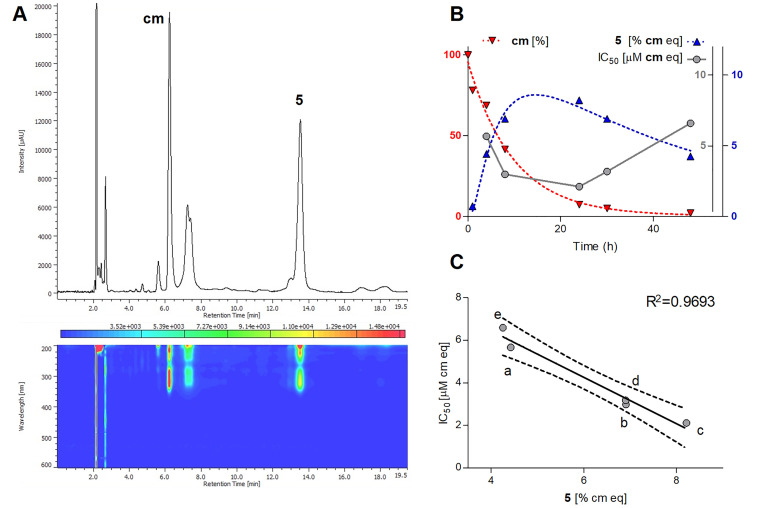
Results of the longitudinal study on the reaction between methyl caffeate (**cm**) and α,α′-azodiisobutyramidine dihydrochloride (AAPH). (**A**) Supercritical fluid chromatography-photodiode array (SFC-PDA) fingerprint of the most potent cytotoxic sample taken at 24 h of reaction time; chromatogram represents maximum absorbance in the wavelength range of λ = 350–600 nm. (**B**) Time dependency of the reaction and the IC_50_ values of the respective samples taken at 0 h (t_0_), 1 h (a), 4 h (b), 8 h (c), 24 h (d), 30 h (e), and 48 h (f) on HeLa cells. Amounts of compound **5** (right y-axis, blue) and IC_50_ values (right y-axis, grey) of the samples are expressed in **cm** equivalents (µM and %, respectively), nonlinear regression for the amounts of **cm** (left y-axis, red) and **5** was performed by the one-phase decay and the log Gaussian models of GraphPad Prism 5.0, respectively. (**C**) Linear correlation between the relative area under the curve (AUC) values of compound **5** and the IC_50_ values. The 95% confidence interval of the regression line is shown with dashed lines; AUC is given in % relative to that of **cm** at t_0_.

To find the best reaction time for the isolation of the cytotoxic constituent(s), a longitudinal study of this reaction was performed. Samples were taken out at specified reaction times (i.e., 0, 1, 4, 8, 24, 30, and 48 h), analyzed for their product profiles by HPLC and SFC, and tested for their cytotoxicity in parallel with the analytical measurements. As the reaction proceeded and the amount of **cm** decreased, an expected increase in the amounts of the products was observed, that was accompanied by a gradual increase in the cytotoxic effect until 24 h. After that time, the bioactivity started to gradually decrease. Results of this study along with the SFC fingerprint of the most potent sample are presented in Figure 3.

### 3.3. Bioactivity-Guided Isolation of the Cytotoxic Metabolite of cm

**Figure 4:**
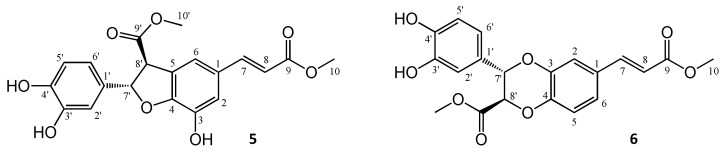
Structures of oxidized products isolated from the reaction of methyl caffeate (**cm**) with AAPH. For both compounds **5** and **6**, only one enantiomer is shown for clarity.

The reaction monitoring gave an excellent correlation between the amounts of compound **5** present in the samples and their cytotoxic activity on HeLa cells (Figure 3C). The curve fitting did not predict a significantly higher maximum yield for this compound at another reaction time than that observed experimentally at 24 h, therefore this reaction time was selected for a scale-up to isolate compound **5**. To enable the isolation of further active compounds, a bioactivity-guided purification strategy was chosen. The reaction mixture was separated to main fractions by preparative HPLC, and all fractions were tested for their cytotoxic activity on HeLa cells. Fraction 3, containing mainly compound **5**, exerted ~80% inhibition at 1.8 µM concentration as expressed in **cm** equivalents. Other fractions were found essentially inactive in the cell viability assay, but fraction 5 contained a major product that was therefore also selected for isolation. Accordingly, two compounds (**5** and **6**) were isolated from fraction 3 and 5, respectively. Their structures (Figure 4) were elucidated by 1- and 2D NMR spectroscopy.

The NMR chemical shifts showed good agreement with those published in the literature for both compounds **5 [15]**, and **6 [29]**. Both oxidized derivatives are dimerization products of **cm**. The cytotoxic activity of the purified compound **5**, a dihydrobenzofuran lignan, was investigated on a panel of human gynecological cancer cell lines (HeLa, SiHa, MCF-7, MDA-MB-231); the results are shown in Table 1.

It is known that polyphenolic compounds can react with cell culture reagents to generate H_2_O_2_ that may produce bioactivities erroneously assigned to the polyphenol itself [30], but this effect may be of little importance here. Compound 5 bears a catechol group; therefore, it may be considered as an “antioxidant” itself, somewhat similarly to its (non-cytotoxic) parent compound **cm**. However, antitumor properties of compound 5 have been extensively studied both in vitro and in vivo [17], therefore its pharmacological value may be considered as well established. Unlike a simple cytotoxic compound, compound **5** is a highly promising lead compound for further clinical development based on its potent antiangiogenic activity [16] and particularly its IL-25 secretion-increasing effect on tumor-associated fibroblasts, that manifests in an in vivo antimetastatic activity at a dose as low as 20–100 µg/kg [17]. It is of interest that compound **5** was also present in the metabolite mixture obtained from the reaction of **cm** with peroxynitrite (see Supplementary Materials, Figures S1, S5, and S6). Considering the abundant presence of peroxynitrite in various tissues and its complex, critical role in redox signaling and oxidative stress [31,32], it is a very interesting finding to observe the formation of a highly potent antitumor compound (such as **5**) from a dietary antioxidant (such as **cm**) upon scavenging this RNS.

**Figure 5:**
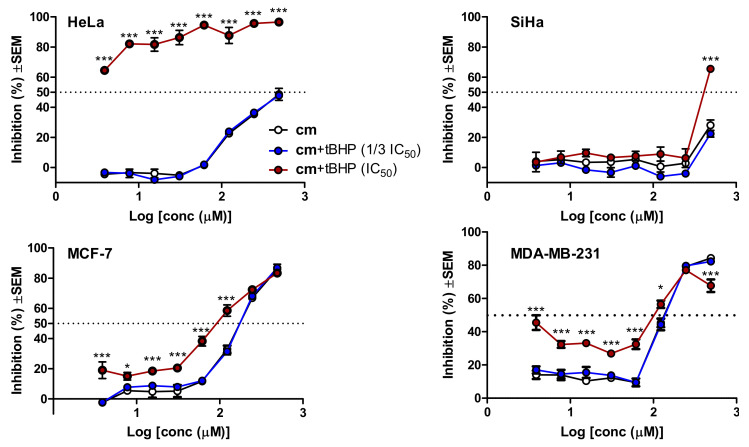
Cytotoxic activity of methyl caffeate (**cm**) on gynecological cancer cells with or without *tert*-butyl hydroperoxide (*t*-BHP)-induced intracellular oxidative stress. Cells were pre-treated with *t*-BHP for 24 h at its 1/3 IC_50_ or IC_50_ concentration that was previously determined in a 72 h MTT assay, then the medium was removed, the cells were washed with PBS, and **cm** treatment was performed for 72 h in freshly added media. Results were analyzed by two-way ANOVA followed by a Bonferroni post-hoc test, * and ***: *p* < 0.05 and *p* < 0.001, respectively, as compared to the single treatment with **cm**; *n* = 3.

Subsequently, it was also our aim to evaluate whether a possible oxidative stress-related intracellular in situ formation of compound **5** (and possibly other bioactive metabolites) from **cm** has the chance to modulate the observed cytotoxic effect. To explore this, we first determined the cytotoxicity of *tert*-butyl hydroperoxyde (*t*-BHP), a well-known lipophilic inducer of intracellular oxidative stress [33], in a 72 h MTT experiment on each cell line. This resulted in IC_50_ values of 27.1, 23.0, 11.3, and 7.9 µM for HeLa, SiHa, MCF-7 and MDA-MB-231, respectively (*n* = 6), i.e., the four cell lines showed only moderate differences in their sensitivity to the cytotoxic effect of *t*-BHP. In a recent in-depth longitudinal study it was shown that a 24 h treatment with *t*-BHP induced significant levels of oxidative stress in MCF-7 cells [33]. Based on this, and to prevent a chemical reaction between *t*-BHP and **cm** that could possibly be catalyzed by cell culture medium components [30], we planned a two-step combination experiment where the cells were pre-treated with *t*-BHP at 1/3 IC_50_^(72 h)^ or IC_50_^(72 h)^ concentrations for 24 h, then the medium was removed, the cells were washed, and then treated by **cm** in freshly added media. Cell viability was then assessed after 72 h, and inhibitions were calculated based on cell controls that were subjected to the same treatment but without the addition of **cm** in the second medium. Results are shown in Figure 5. 

With the sole exception of SiHa cells that are also the most resistant to the activity of compound **5**, all other cell lines were sensitized to the cytotoxicity of **cm** by the oxidative stress-inducing effect of *t*-BHP. It is also noteworthy that by far the largest increase in the killing activity of **cm** took place in HeLa cells, where the difference between **cm** and its most active hypothesized metabolite was also much larger than in the other cell lines. Because the lower concentration *t*-BHP pre-treatment did not exert any significant change in the killing activity of **cm** on any of the cell lines at any activity levels, it is unlikely that we were experiencing an artefact due to the reaction between remaining traces of extracellular *t*-BHP and **cm**. To double-check this, we also performed checkerboard combination experiments using simultaneous co-treatment with *t*-BHP and **cm**, and antagonism was observed in each cell line; e.g., in HeLa cells a 50% combination index (CI_50_) value of 4.75 was calculated by the Chou method [34], demonstrating a strong antagonism. Therefore, the increased bioactivity of **cm** in *t*-BHP-pre-treated cells was not due to a chemical reaction between these two agents, but perhaps due to the increased intracellular oxidative stress. Nevertheless, the increased efficiency of **cm** in such conditions could be due to many reasons, among which the alleged formation of bioactive metabolites (e.g., compound **5**) is only one possibility. The complex situation is well shown by the case of the MDA-MB-231 cells (Figure 5) where, in contrast with the single treatment, the cytotoxicity of **cm** on the highly stressed cells could not be clearly described with a single sigmoidal dose–response curve.

We also attempted to conduct HPLC-MS/MS studies on lysates of cells pretreated similarly, but these did not lead to conclusive results. Therefore, direct evidence to the biological relevance of an ROS/RNS scavenging-induced transformation of **cm** to compound **5** is currently not available.

## 4. Conclusions

This work aimed to evaluate the possible pharmacological value of product mixtures obtained by chemical reactions between an antioxidant (i.e., **pcm** and **cm**) and fully biorelevant ROS/RNS-modeling chemical systems. This approach led to the successful identification of an active metabolite that is a highly potent antitumor lead (i.e., compound **5**), previously shown to act as an antimetastatic agent through a unique mechanism of action. Furthermore, we found that pre-treatment of cancer cells with *t*-BHP, a well-known inducer of intracellular oxidative stress, modulated the cytotoxicity of **cm** in a way that coincided with the cells’ sensitivity to this RONS scavenging-related metabolite.

Altogether, our results provided direct evidence for the RONS-mediated transformation of an abundant dietary antioxidant into a potent antitumor agent. At this point, no conclusion can be drawn about the rate by which this may actually happen in a biological environment under oxidative stress, and therefore it is unknown what would be the relevance of this phenomenon in terms of the chemical biology behind the bioactivity of **cm**. Nevertheless, this work provides a proof-of-concept to our initial working hypothesis, i.e., that oxidative stress-related metabolite patterns of small-molecule natural antioxidants serve as a rich pool of bioactive compounds with a high value for drug discovery.

## Figures and Tables

**Table 1:** Cytotoxic activity of compound **5** in comparison with its parent compound **cm**. Cisplatin was used as positive control; results were obtained from two biological replicates, 5 replicates each (*n* = 10); 95% C.I. refers to 95% confidence interval for the calculated IC_50_ values.

Compound	IC_50_ [95% C.I.] (µM)			
	HeLa	SiHa	MCF-7	MDA-MB-231
**cm**	450 [396.7–551.2]	>500	175.4 [162.3–189.7]	139.3 [116.5–166.6]
**5**	1.1 [1.0–1.2]	>30	1.1 [0.9–1.4]	3.9 [3.1–4.9]
cisplatin	11.7 [10.3–13.1]	13.6 [12.6–14.7]	5.2 [4.6–5.8]	25.8 [24.4–27.4]

## Supplementary Materials

The following are available online at https://www.mdpi.com/2218-273X/10/11/1537/s1, Figure S1: Chromatographic fingerprint of the continuous-flow reaction of **cm** with peroxynitrite (C6), Figure S2: Chromatographic fingerprint of the reaction of **cm** with AAPH in acetonitrile:water (1:1, *v/v*), Figure S3: Chromatographic fingerprint of the reaction of **cm** with AAPH in acetonitrile:water (9:1, *v/v*), Figure S4: Chromatographic fingerprint of the reaction of **cm** with AAPH in methanol:water (1:1, *v/v*), Figure S5: Mass spectrum of compound **5** within the oxidized mixtures at Rt = 7.49–7.51 min, Figure S6: SFC-PDA fingerprint of the reaction of **cm** with AAPH at its maximum yield of compound **5** (**A**) in comparison with that of the reaction of **cm** with peroxynitrite (**B**), and UV spectra of the peaks corresponding to compound **5**, Table S1: Cytotoxic activity of the above reaction mixtures against human gynecological cancer cell lines in comparison with the parent compound **cm**.
