# Supplementary material for: AAPH or Peroxynitrite-Induced Biorelevant Oxidation of Methyl Caffeate Yields a Potent Antitumor Metabolite
Source: Biomolecules. 2020 Nov 11;10(11):1537. doi: 10.3390/biom10111537 (PMC7697082; doi:10.3390/biom10111537)
Supplement: Supplementary file 1 [file biomolecules-10-01537-s001.pdf]

Figure S1. Chromatographic fingerprint of the continuous-flow reaction of **cm** with peroxynitrite (C6). DAD: diode array detection, λ= 280$\pm$7nm, BPC: Base peak chromatogram. Analysis was performed on a Cortecs (C18, 150 x 4.6 mm, 2.7 µm) column with a gradient elution of Solvent B (0.1 % TFA in acetonitrile:water / 95:5) in Solvent A (0.1 % TFA in H_2O) from 0 to 100 % in 10 minutes, and washed with 100 % B from 10 to 12 minutes.

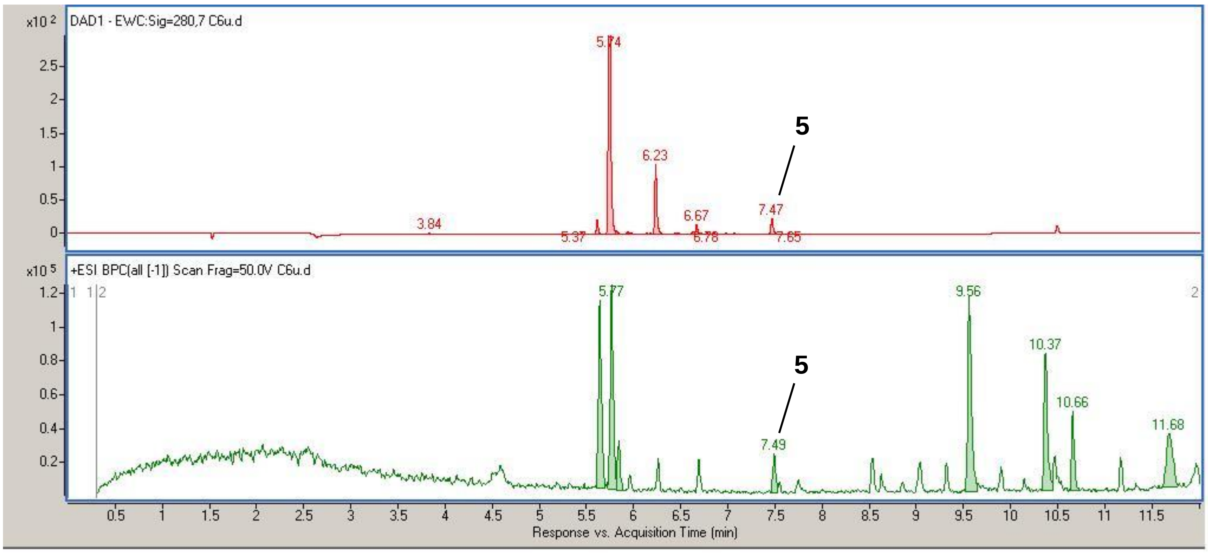

Figure S2. Chromatographic fingerprint of the reaction of **cm** with AAPH in acetonitrile - water (1:1, v/v). DAD: diode array detection, λ= 280$\pm$7nm, BPC: Base peak chromatogram. Analysis was performed on a Cortecs (C18, 150 x 4.6 mm, 2.7 µm) column with a gradient elution of Solvent B (0.1 % TFA in acetonitrile:water / 95:5) in Solvent A (0.1 % TFA in H_2O) from 0 to 100 % in 10 minutes, and washed with 100 % B from 10 to 12 minutes.

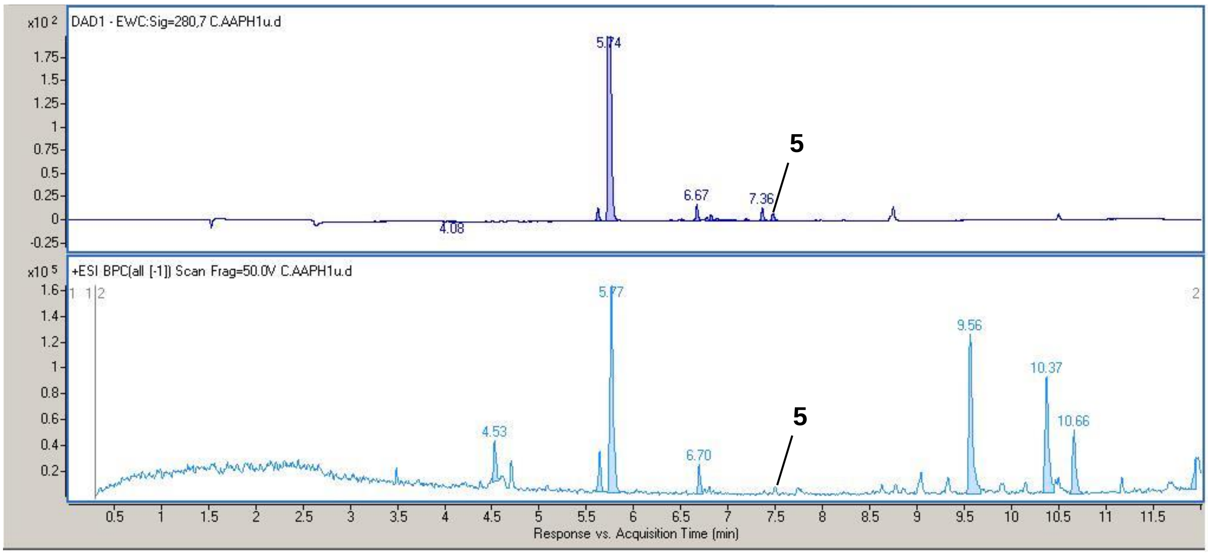

Figure S3. Chromatographic fingerprint of the reaction of **cm** with AAPH in acetonitrile - water (9:1, v/v). DAD: diode array detection, λ= 280±7nm, BPC: Base peak chromatogram. Analysis was performed on a Cortecs (C18, 150 x 4.6 mm, 2.7 µm) column with a gradient elution of Solvent B (0.1 % TFA in acetonitrile:water / 95:5) in Solvent A (0.1 % TFA in H_2O) from 0 to 100 % in 10 minutes, and washed with 100 % B from 10 to 12 minutes.

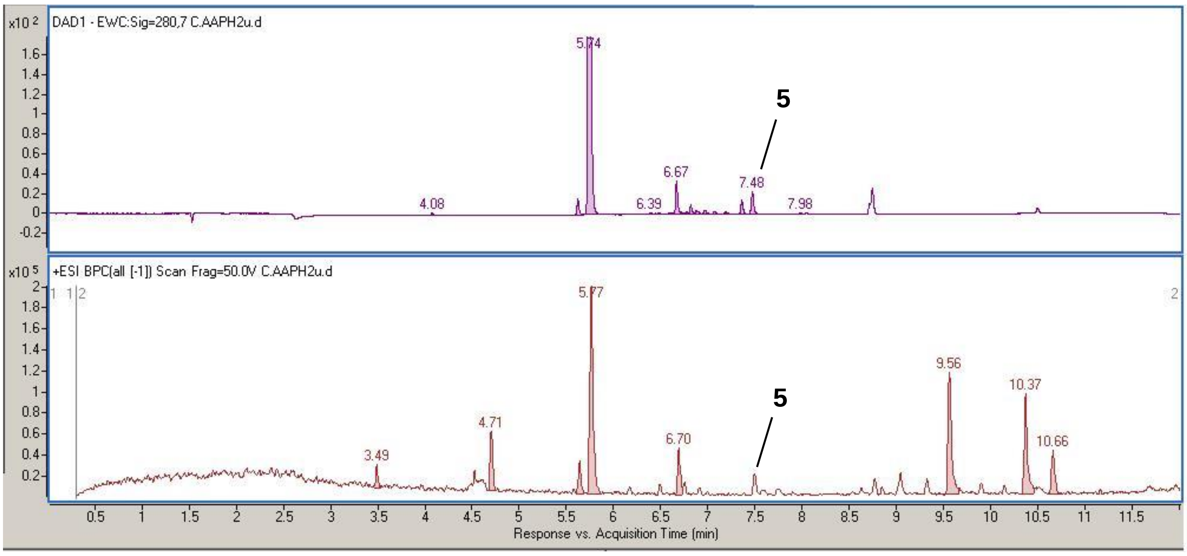

Figure S4. Chromatographic fingerprint of the reaction of **cm** with AAPH in methanol - water (1:1, v/v). DAD: diode array detector, λ= 280±7nm, BPC: Base peak chromatogram. Analysis was performed on a Cortecs (C18, 150 x 4.6 mm, 2.7 µm) column with a gradient elution of Solvent B (0.1 % TFA in acetonitrile:water / 95:5) in Solvent A (0.1 % TFA in H_2O) from 0 to 100 % in 10 minutes, and washed with 100 % B from 10 to 12 minutes.

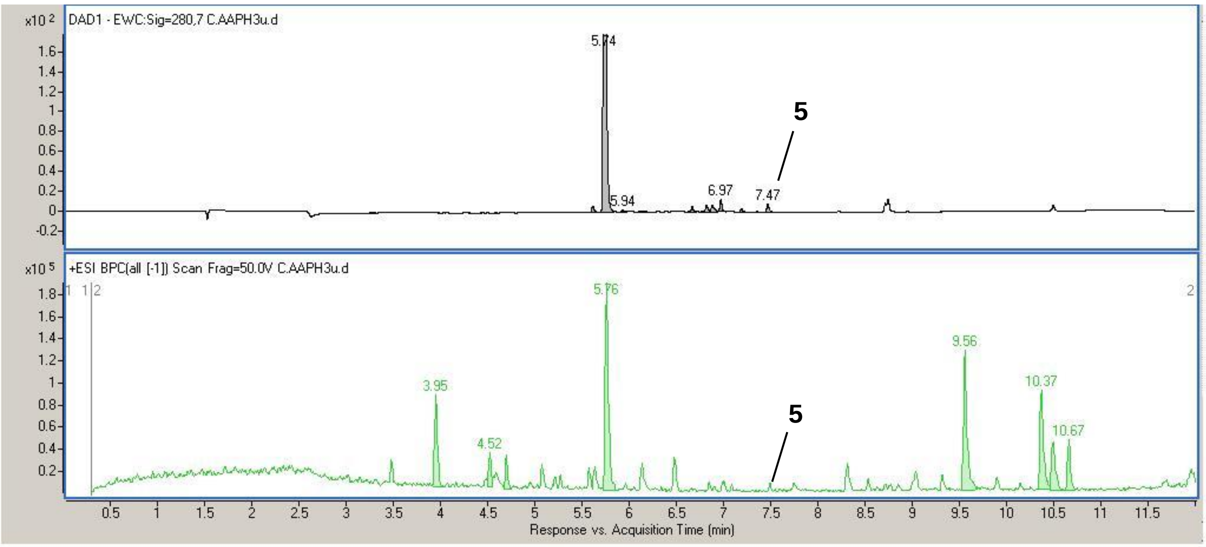

Figure S5. Mass spectrum of compound **5** within the oxidized mixtures at Rt=7.49-7.51 min. Base peak m/z=387.1

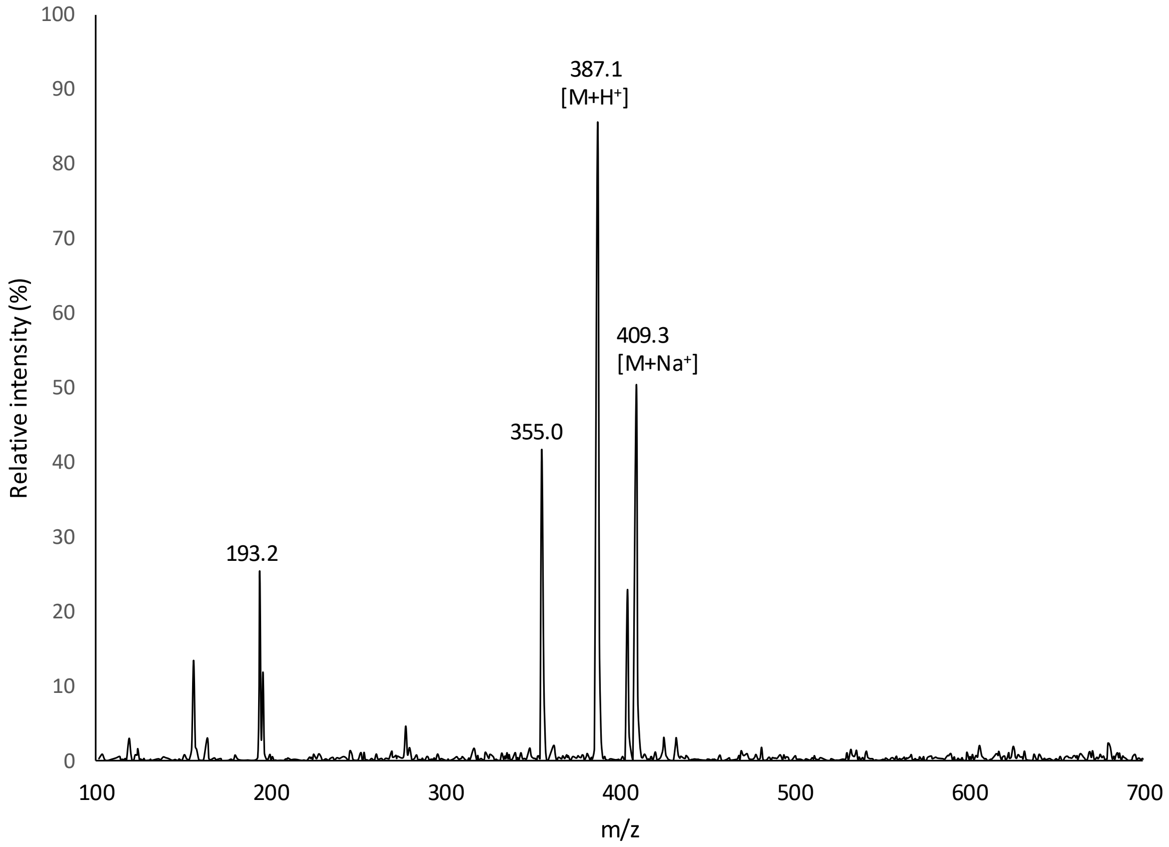

Figure S6. SFC-PDA fingerprint of the reaction of **cm** with AAPH at its maximum yield of compound **5** (**A**) in comparison with that of the reaction of **cm** with peroxynitrite (**B**), and UV spectra of the peaks corresponding to compound **5**. These provide an independent proof for the peroxynitrite scavenging-related formation of compound **5** from **cm**.

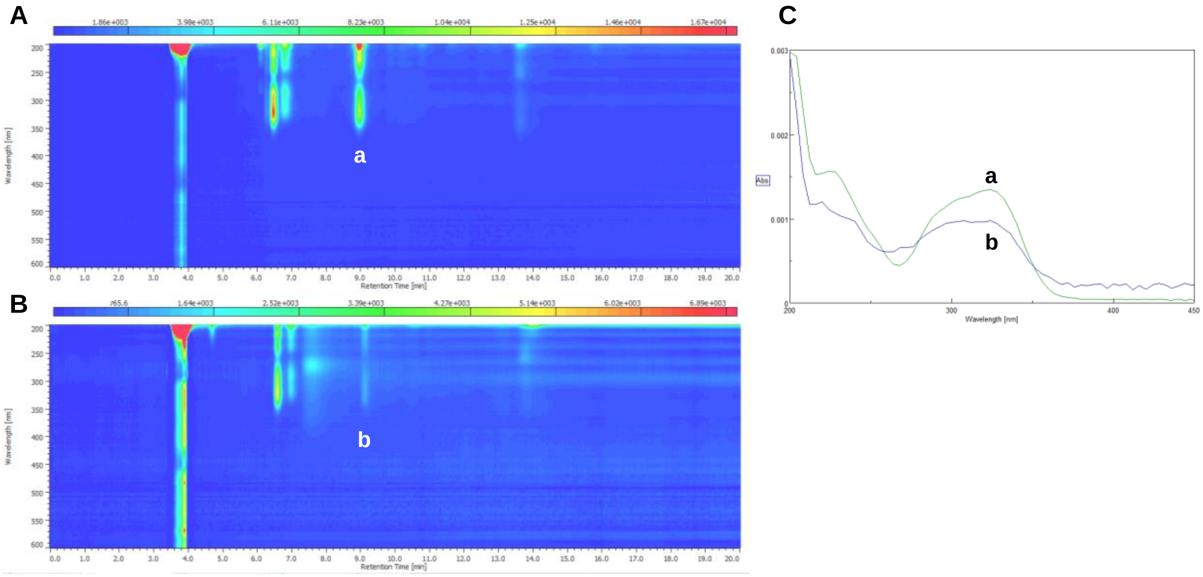

Table S1. Cytotoxic activity of the above reaction mixtures against human gynecological cancer cell lines in comparison with the parent compound **cm**. Concentrations are 10 or 30 µM in **cm** equivalents, i.e. dilutions of each product mixture were performed as for **cm**. Results were obtained from 5 parallel measurements. Sample codes represent sample numbers of the above chromatographic fingerprints. All reaction mixtures obtained analogously from **pcm** exerted below 20% inhibitions at 10 and 30 µM, therefore they are not presented here.

Sample	Concentration (µM **cm** equiv.)	Inhibition ± SEM (%)			
		HeLa	SiHa	MCF-7	MDA-MB-231
**cm**	10	< 20	< 20	< 20	< 20
	30	< 20	< 20	28.6 ± 0.5	< 20
**C6**	10	23.1 ± 0.9	< 20	< 20	< 20
(Fig. S1)	30	72.8 ± 0.4	< 20	53.0 ± 1.0	39.6 ± 1.2
**C.AAPH1**	10	< 20	< 20	< 20	< 20
(Fig. S2)	30	80.7 ± 0.9	< 20	57.5 ± 2.2	40.6 ± 1.5
**C.AAPH2**	10	49.9 ± 1.1	< 20	42.8 ± 1.0	32.8 ± 2.0
(Fig. S3)	30	84.0 ± 0.6	29.8 ± 2.4	63.1 ± 0.6	41.8 ± 0.9
**C.AAPH3**	10	< 20	< 20	< 20	< 20
(Fig. S4)	30	26.1 ± 2.8	< 20	26.7 ± 2.6	< 20
